# Evolutionary mechanism of the defects in the fluoride-containing phosphate based glasses induced by gamma radiation

**DOI:** 10.1038/srep18926

**Published:** 2016-01-06

**Authors:** Pengfei Wang, Quanlong He, Min Lu, Weinan Li, Bo Peng

**Affiliations:** 1 State Key Laboratory of Transient Optics and Photonics, Xi’an Institute of Optics and Precision Mechanics, Chinese Academy of Sciences (CAS), Xi’an 710119, P.R. China; 2 University of Chinese Academy of Sciences (UCAS), Beijing 100049, P.R. China

## Abstract

In the laser driven inertial confinement fusion (ICF) experimental target chamber, like the 3ω (351 nm) laser irradiation, the irradiation of gamma ray and X-rays, will also cause the formation and increase of various defects in the investigated series of fluoride-containing phosphate based glasses that have potential use in novel high performance color separation optics. The induced defects contribute to the increase of absorption in the UV region, which will make the UV performance of these laser glasses deteriorated. Some of the induced defects can be bleached to some extent through the subsequent thermal treatment process, resulting from the release and capture of the electrons in conduction band. Through the gamma radiation and post-heat treatment experiments, a general model of the evolutionary mechanism of the defects in these fluoride-containing phosphate based glasses was proposed.

In order to improve the properties of optical materials for high power laser system, stronger lamps, lens system in UV microlithography equipments and other special UV optics, optical glass including fluoride glasses, phosphate glasses and silicate glasses, as well as borate glasses were intensively studied in the last few decades^1^,^2^,^3^. And phosphate based glasses including fluorophosphate glasses have received more attention due to their excellent properties such as high transparency, glass forming ability and good host materials for dopant ions, as compared with other glass systems^4^. As they fulfill the requirement of increasing demands of the high performance UV transmitting materials to be applied for high power lasers, requiring high UV transparency and high pulse laser-induced damage thresholds (LIDTs). Nevertheless, various defects may form in the phosphate based glasses during the glass preparation process, which are harmful for their UV transmittance and significantly decrease their LIDTs at the short wavelength^5^. In that case, it is worthwhile to study the defect-state both in these as-produced glasses and the in-use ones for the purpose of enhancing their UV performance.

The defects formed in the phosphate based glasses generally can be divided into two types, the intrinsic defects that arise from the raw materials and glass matrix and the extrinsic defects which are caused by dopants or impurities^3^,^6^. As we all know, phosphate based glasses typically consist of phosphate chains which is made of P-tetrahedra. The modifier cations^2^, the glass preparation process^3^,^4^ including but not limited to glass melting and thermal annealing conditions, as well as energetic radiations^1^,^4^, may have influence on the breakage of the phosphate chains, which subsequently form multiple intrinsic defects, including phosphate-related oxygen hole center (POHC), oxygen-related hole center (OHC), and phosphate-related electron centers (PEC) including PO_2_-EC, PO_3_-EC and PO_4_-EC defects^5^,^7^,^8^, etc. On the other hand, some unavoidable trace impurities such as iron and other intentionally or unintentionally doped transition metals could produce numerous extrinsic defects^9^. Many studies^2^,^3^,^4^ have been carried out on these defects associated with transparency for developing more efficient and high transmittance materials to improve the performance of the corresponding devices. Besides the high power UV laser irradiation, potential use of the series of fluoride containing phosphate based glasses in Megajoule class lasers as the National Ignition Facility (NIF) in the USA and SG III in China^5^ will also suffer from the irradiation of the gamma rays and X-rays in the experimental chamber^10^. Unfortunately, the nature of the defects in these specific glasses is very complex and still not explicit, especially for the evolutionary mechanism of the produced defects.

Therefore, to explore the information about the defects is critical for understanding of their evolutionary mechanism. In this work, we investigated the types of defects in fluoride containing phosphate based glasses that were prepared in a special reducing atmosphere through analysis of the absorption spectra, Raman spectra and electron paramagnetic resonance (EPR) spectra. Besides, a model of the evolutionary mechanism of these defects produced by the gamma radiation and their transformation via a subsequent thermal treatment process was finally proposed based on the systematic analysis.

## Results

### The influence of gamma radiation on the defects and corresponding spectral property of the series of fluoride-containing phosphate based glasses

**Figure 1:**
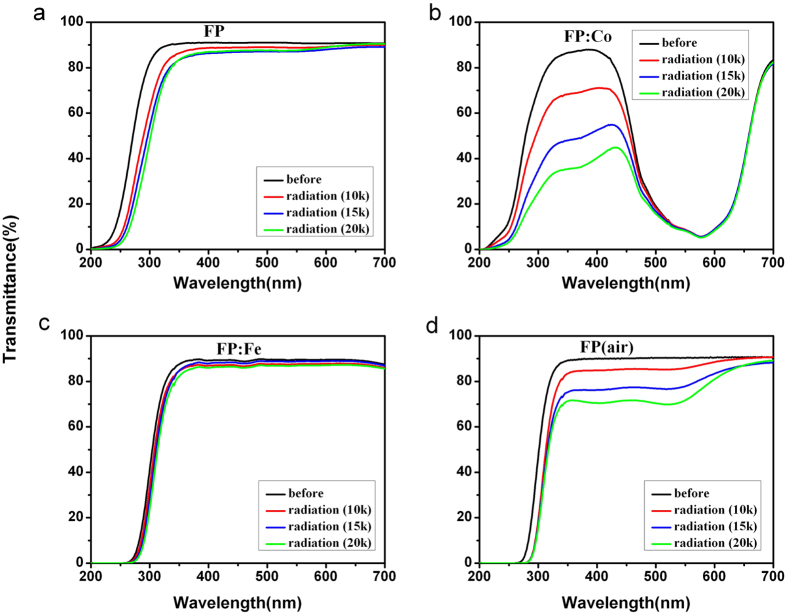
The UV/VIS transmission spectra of FP, FP: Co, FP: Fe and FP (air) glass samples (thickness 10 mm) before and after gamma radiation (10, 15 and 20 krad (Si), respectively).

Figure 1 shows the optical transmission spectra of the series of glass samples before and after gamma radiation (10, 15 and 20 krad (Si), respectively). All the samples show high transparency in the range of 350–700 nm before gamma radiation, except for the FP: Co glass sample which presents the low transmittance in the range of 440–670 nm resulting from the absorption of cobalt ion. Gamma radiation lead to an obvious decline of the transmittance in the UV and visible spectrum range, especially for FP: Co glass sample as shown in Fig. 1b. The descending range of the transmittance in 200–500 nm could be described as FP: Co > FP (air) > FP > FP: Fe, which might be associated with the concentration of various radiation-induced defects in these glasses. Besides, it can be seen that gamma radiation causes some degree of red-shift of the absorption edge as compared with the ones without being irradiated. This phenomenon is related to the incremental concentration of Fe^3+^ and other radiation-induced defects which have large absorption in the short wavelength region^8^.

**Figure 2:**
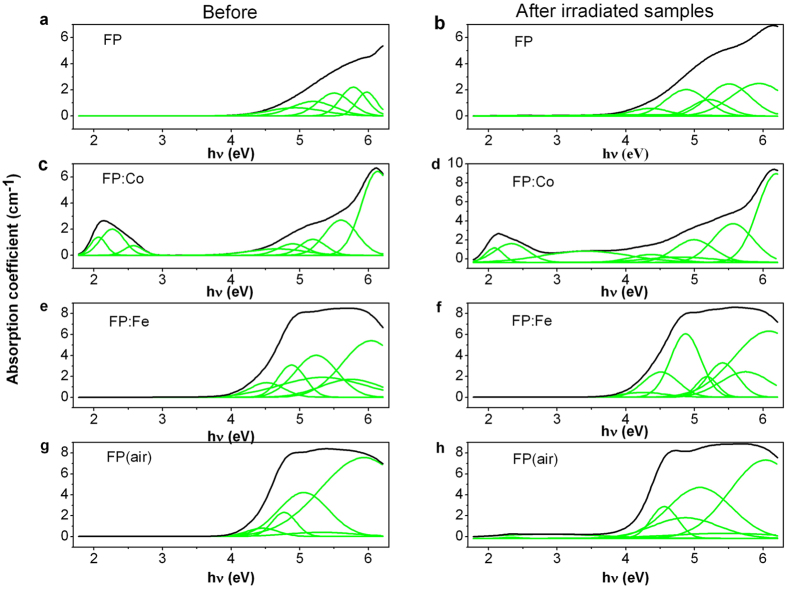
The absorption spectra including band separation of FP, FP: Co, FP: Fe and FP (air) glass samples (thickness 10 mm) before and after gamma radiation (20 krad (Si)).

In Fig. 2 the absorption spectra including band separation demonstrates that several types of defects were generated in the series of phosphate based glasses under gamma radiation with a dose of 20 krad (Si). The separated absorption bands identified through the Gaussian peak fitting that corresponds to various defects are given in Table 1. It can be found that the bands of POHC lie in the low energy region, and several bands connected with PO_3_-EC, PO_4_-EC, PO_2_-EC^8^, and (Fe^2+^)^+^ defects are positioned near the high energy region^9^. The bands located at around 5.5 eV are associated with the fluorine-related non-paramagnetic color centers (FD centers, with a similar structure of STE^11^). A band actually consisting of three superposed peaks at around 2.13 eV was also observed for the FP: Co sample, as shown in Fig. 2c, which could be assigned to the absorption of Co^2+^ and POHC defects. It can be noticed that the absorption peak of POHC defect disappeared when exposed to gamma radiation. Meanwhile, a new band (at 3.4 eV) related to (Co^2+^)^+^ was found after radiation, which is due to Co^2+^ was photo-oxidized to (Co^2+^)^+^. Besides, an additional peak (at around 4.3 eV) arising from OHC defect was also observed in irradiated glasses except for FP (air) sample.

**Figure 3:**
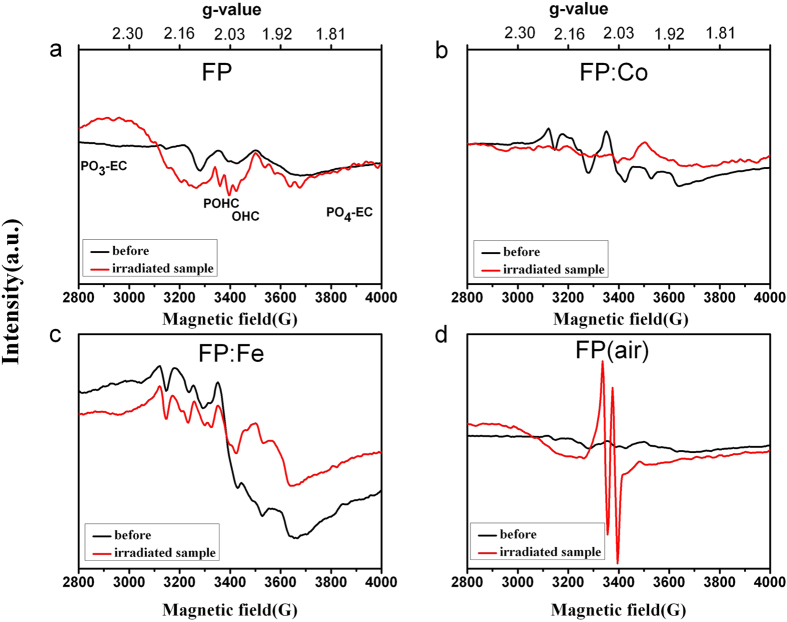
The EPR spectra of FP, FP: Co, FP: Fe and FP (air) samples before and after gamma radiation with a dose of 20 krad (Si).

Typical EPR spectra of the above samples before and after gamma radiation are compared in Fig. 3. All EPR spectra of the undoped and doped glasses are dominated by the signals of intrinsic defects. In phosphate based glasses the main signal is the POHC defects with the g-value around 2.0^6^,^11^. This signal was significantly enhanced in the doped glass samples. After gamma radiation the signals become apparent in the undoped glasses, especially for the FP (air) sample, while it is weaker in Co and Fe single-doped samples. Another signal located at side of POHC signal could also be found in the irradiated samples except for the FP (air) sample, which is associated with the OHC defects caused by gamma radiation. It can be noticed that the signals of PO_3_-EC and PO_4_-EC become enhanced for both the FP and FP (air) glass samples, while becoming less intense in Fe and Co doped samples as compared with that of the unirradiated samples. These results suggest that gamma radiation causes much more defects in glass, while Co and Fe ions could suppress the formation of POHC, PO_3_-EC and PO_4_-EC defects.

**Figure 4:**
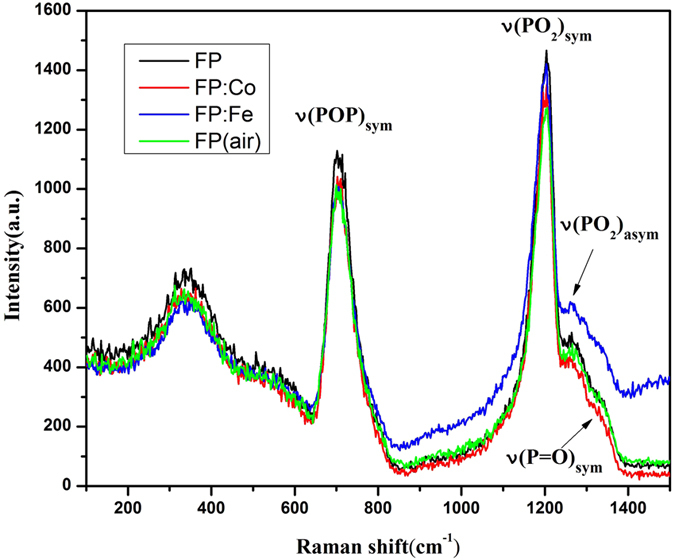
Raman spectra of irradiated FP, FP: Co, FP: Fe and FP (air) samples with a gamma radiation dose of 20 krad (Si).

The strongest peaks are located at around 1202 cm^−1^ arising from the symmetric resonances (O-P-O) of the non-bridging oxygens on the PO_2_ of phosphate chain as shown in Fig. 4. The other dominant peaks at 706 cm^−1^ are associated with a symmetric stretching (P-O-P) mode of bridging oxygens of Q^2^ units^12^. Apart from these pronounced peaks there are several less pronounced bands in the spectra. The bands cover a broad region (200–600 cm^−1^) originating from the internal deformation bending modes of PO_2_ and O-P-O chain. And the weak bands were also observed in the region of 870–970 cm^−1^, especially for the FP: Fe sample, which is due to the end-group dynamics of the phosphate chain. Besides, two small bands as the shoulders of the peak of 1202 cm^−1^ at 1262 cm^−1^ and 1338 cm^−1^ are assigned to asymmetric stretching of non-bridging oxygens and symmetric stretching of doubly-bonded oxygen (P=O) characteristic of a three-dimensional cross-linked structure, respectively^13^.

### The influence of heat treatment on the defects absorption and concentration level in the series of fluoride-containing phosphate based glasses

**Figure 5:**
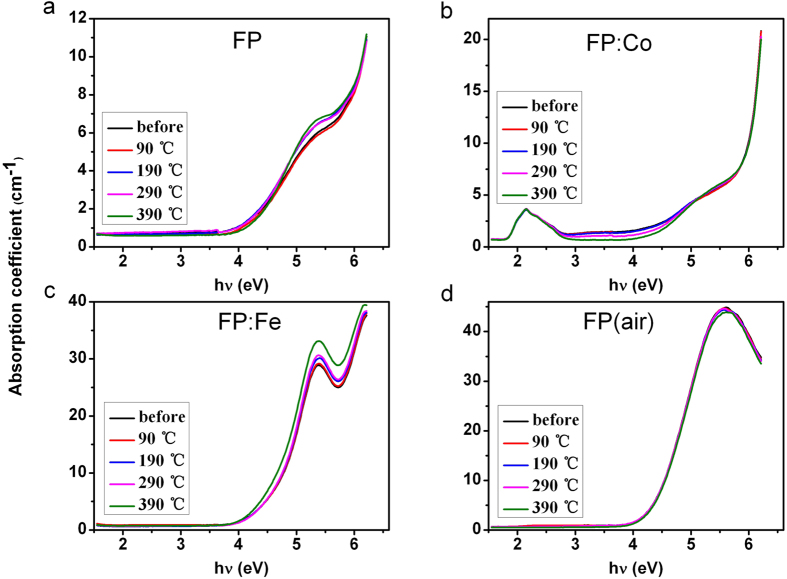
Absorption spectra of FP, FP: Co, FP: Fe and FP (air) samples (thickness 1.5 mm) heat treated at different temperature (90 ^o^C, 190 ^o^C, 290 ^o^C and 390 ^o^C) at ambient conditions.

In order to reveal the evolutionary mechanism of defect, the irradiated samples were also thermal treated at different temperature at ambient conditions, and the change of their induced absorption spectra are compared in Fig. 5. The absorption spectra are analyzed similarly using the Gaussian peak fitting method to get the separated absorption bands corresponding to the above identified defects as shown in Table 1. It can be seen that with increasing heat treatment temperature, the absorption bands decrease at the low energy region, while increase at the high energy region even though the significant difference of the spectral shape exists among the different glass samples, suggesting that the regularity of the evolutionary mechanism.

**Figure 6:**
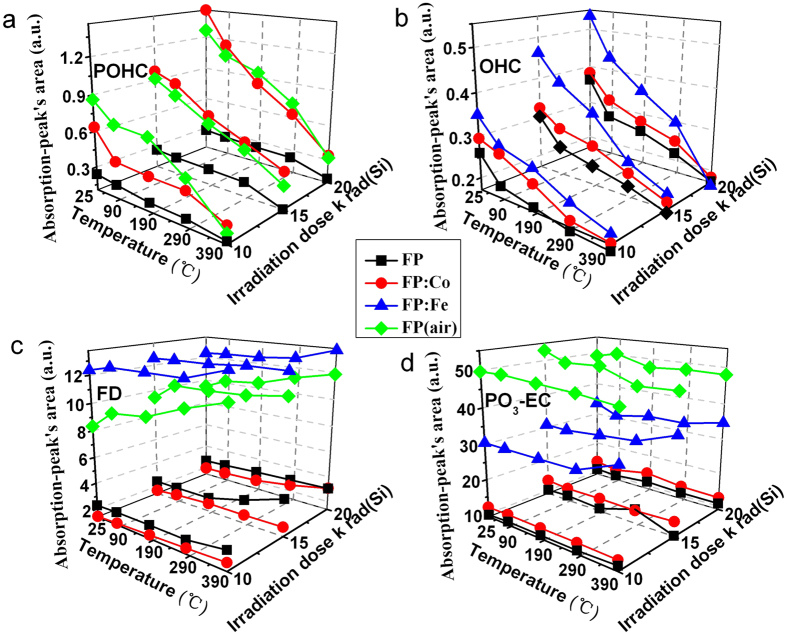
Line chart displaying the absorption-peak’s area of corresponding intrinsic defects in the FP, FP: Co, FP: Fe and FP (air) samples irradiated with different radiation dose (10, 15 and 20 krad (Si)) and heat treated at different temperatures (90 ^o^C, 190 ^o^C, 290 ^o^C and 390 ^o^C).

**Figure 7:**
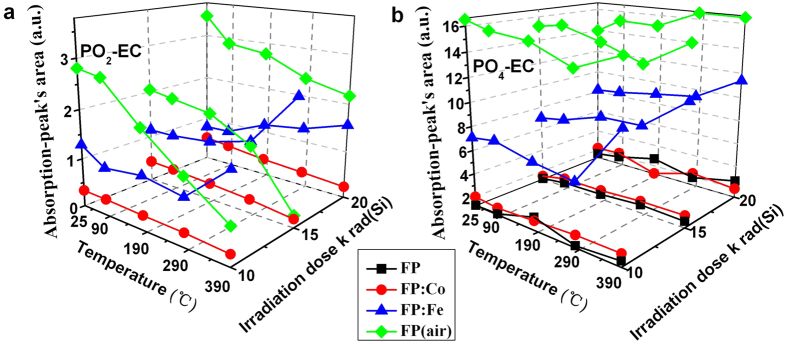
Line chart displaying the absorption-peak’s area of PEC defects in the FP, FP: Co, FP: Fe and FP (air) samples irradiated with different radiation dose (10, 15 and 20 krad (Si)) and heat treated at different temperatures (90 ^o^C, 190 ^o^C, 290 ^o^C and 390 ^o^C).

**Figure 8:**
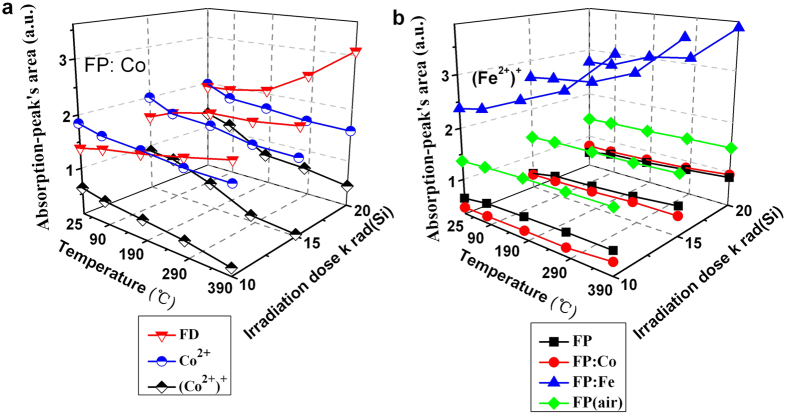
Line chart displaying the absorption-peak’s area of Co^2+^, (Co^2+^)^+^ and FD center defects in FP: Co samples (a) and (Fe^2+^)^+^ defects in FP: Fe samples (b) irradiated with different radiation dose (10, 15 and 20 krad (Si)) and heat treated at different temperatures (90 ^o^C, 190 ^o^C, 290 ^o^C and 390 ^o^C).

Figures 6,7,8 present the variety of the absorption-peak’s area of the corresponding defects with the heat treatment temperatures in order to illustrate the changes in the concentration of various defects in the heat treated glass samples. The concentration of POHC defect is significantly higher in FP: Co and FP (air) samples than in the FP sample, as shown in Fig. 6a. The concentration increases with the increase of radiation doses, while it decreases with the increase of heat treatment temperature. Besides, there is weak dependence on gamma radiation dose by POHC defect in the FP sample, and it is insensitive to temperature variation, especially in low heat treatment temperature range. No POHC defect was found in irradiated FP: Fe sample with the Gaussian peak fitting method but indicated by very weaker signal of its EPR spectrum (Fig. 3c), indicating Fe ions prevented the formation of POHC defects in the phosphate based glass, which is beneficial to the improvement of the radiation resistance of fluoride-containing phosphate based glass materials. In Fig. 6b, it can be seen that there are different concentration levels of OHC defects in those glasses except for the FP (air) sample, and the concentration level could be described as an order of FP: Fe > FP: Co > FP. The concentrations of OHC defects decrease with the increase of heat treatment temperature and the tendency is obvious even though at low heat treatment temperature range. It was also found that the OHC concentration level increases relatively slow with the increase of gamma radiation doses for the FP and FP: Co samples. Fig. 6c shows the changing tendency of the FD defect with different radiation dose and heat treatment temperature for all these four types of investigated samples. In details, the FP: Fe sample maintains the highest FD defect concentration level, followed by the FP (air) sample. The FD defect concentration levels in the above two samples are much higher than that of the FP and FP: Co samples which is very close. It is also illustrated that the FD defect is insensitive to the gamma radiation doses, and its concentration level increases slowly at ≤190 ^o^C, but with further increase of heat treatment temperature, its increasing tendency becomes relatively apparent. As seen in Fig. 6d, the concentration level of PO_3_-EC defect in the FP (air) sample is also twice of the FP: Fe sample and four times higher than that of the FP and FP: Co samples. It can be found that the influences of the gamma radiation doses and heat treatment temperatures on the concentration level of PO_2_-EC defect are not distinct (Fig. 7a) and the same phenomenon could also be found for PO_4_-EC defect (Fig. 7b).

Figure 6c and d indicate FD and PO_3_-EC defects are relatively stable defects in the fluoride-containing phosphate based glasses, which is insensitive to the gamma radiation in the investigated radiation doses range and the heat treatment temperature variations, especially for the PO_3_-EC defect. It might because that the FD and PO_3_-EC defects are mainly associated with the skeleton structure of fluorophosphate glasses. For these investigated fluoride-containing phosphate based glasses, phosphate contents dominate the glass composition, therefore, in these glasses PO_3_-EC will be the main defects, thus it’s concentration level is much greater than that of the FD defect. Combined with the result of upper experiment, we can infer that the POHC and OHC defects are mainly caused by gamma radiation, and it could be effective to reduce these defects in these fluoride containing phosphate based glasses by heat treatment near their transition temperatures.

Besides, the doping of iron ions in these glasses could suppress the formation of POHC defects in glass, while increase the formative probability of FD and PO_3_-EC defects which is closely related to the glass skeleton. These results suggest that Fe ions maybe enter the glass network of fluoride-containing phosphate based glass and strengthen the glass skeleton structure, which results in the decreased number of non-bridging oxygen and conduce to the decreased concentration level of POHC defect that is closely related to non-bridging oxygen. As for the dopant Co ions, its doping concentration is much lower as compared to that of the iron, thus its effect on the glass skeleton structure and the formation of POHC is relatively weaker as compared with the dopant iron ions. In addition, it is the reducing atmosphere that contributes to the decrease of the above mentioned various defects in these fluoride-containing phosphate based glasses. It is well known that OHC is one kind of oxygen deficient defect^7^,^14^, which is easily produced under the reducing atmosphere. It is why no OHC defects are found in the FP (air) samples either by the EPR or through their absorption spectra analysis.

Figure 8a shows the changing trends of the concentration levels of the Co^2+^ and (Co^2+^)^+^ defects, as compared with the FD center defects. The concentration level of Co^2+^ and (Co^2+^)^+^ defects decreased a bit with the increase of heat treatment temperatures, meanwhile, the concentration of FD center defects show a rather rapid increase, especially when the heat treatment temperatures are relatively higher (>190 ^o^C). Based on all the above systematic analysis results, we speculate that FD center defects replace extrinsic Co^2+^ and (Co^2+^)^+^ defects against the thermal annealing processing.

With regards to all these four samples (see Fig. 8 b), the concentration of (Fe^2+^)^+^ defect generally increases with increasing heat treatment temperatures. It seems most obvious for the FP: Fe sample, for which a rather high content of iron ions were intentionally doped, even tiny part of Fe^2+^ in the FP: Fe sample was oxidized to Fe^3+^ during the heat treatment process as compared with the other three types of samples that Fe^3+^ were contained as impurity introduced by the raw materials. And the released electrons were then captured by OHC defects and contribute to the decreased concentration of OHC defects.

## Discussion

It is known, the structure of phosphate glasses is networked with a series of long phosphate chains made of phosphate tetrahedra. With regards to these investigated phosphate based glasses in this work, the introduction of alkali (R^+^) and alkaline earth metal cations (R^2+^), i.e. Li^+^, K^+^, Mg^2+^ and Ba^2+^ contribute to the breakage of pure phosphate chains and form various numbers of bridging oxygen atoms connected with P, such as Q^0^, Q^2^ and Q^3^ units^15^ in the as-produced glasses. Although Raman spectra indicate the series of glasses are based on Q^2^ tetrahedra, Q^0^ and Q^2^ units are also existing in these glasses according to van Wazer’s reorganization theory^16^. Modifying metal cations and the reducing glass melting atmosphere cause the connectivity of these units to be reduced and thus defects like structures containing isolated phosphate anions are formed. These defects, including PO_3_-EC, PO_4_-EC and PO_2_-EC defects have large absorption in the short wavelength region as shown in Figs 1 and 2.

The transmission spectra and absorption spectra of irradiated glasses indicate more PO_3_-EC, PO_4_-EC and PO_2_-EC defects were formed during gamma radiation process. More POHC defects were generated in the FP (air) sample. And the weak signal of POHC was observed in FP: Fe glasses indicate iron can suppress the formation of POHC defects. Besides, new defects (i.e. OHC) were generated for the glasses melted in the reducing atmosphere. This suggests that the OHC defects are sensitive to both the glass melting atmosphere and gamma radiation. There is almost no or very low content of POHC defect found in the FP: Fe samples (before and after radiation) indicates the doping of iron ions to some extent suppress the formation of POHC defects in glass, which might be a beneficial method for improving the radiation resistance of the glass.

As a result, it is speculated that OHC defects are located at a deeper energy level at forbidden band of the series of phosphate based glasses compared with POHC defects. With regard to the extrinsic defects, iron and cobalt can be stable in the divalent charge state in these phosphate based glasses^1^. Divalent irons are more easily photo-oxidized to trivalent when the glass samples are exposed to the gamma radiation, which have large absorption at UV region and result in the red-shift of the absorption edge. Figs 5 and 6 indicate the heat treatment at the ambient atmosphere contributes to the healing behavior of gamma radiation-induced POHC and OHC defects. It is no doubt that tiny amount of Fe^2+^ were oxidized to (Fe^2+^)^+^ under the ambient conditions, thus the released electrons were subsequently captured by POHC and OHC defects and then transformed into Q^3^ and other phosphate related units^17^, which result in the decrease of POHC and OHC defects. A weak, but significant transformation of intrinsic POHC into (Fe^2+^)^+^ HC and (Co^2+^)^+^ HC can be deduced from the change of POHC and the extrinsic HC concentration level as shown in Figs 5, 6 and 8. Similar results can also be found in ref.^18^ for the fluoride-phosphate glasses that have a much higher content of fluoride.

The slight decrease in the concentration levels of intrinsic PEC (PO_2_-EC, PO_3_-EC and PO_4_-EC) defects with the increase of heat treatment temperatures is associated with the repair of the P-O bonds in glasses during the heat treatment process at ambient conditions. However, it is not applicable for FP: Fe sample that shows an increase tendency with regard to the concentration levels of PEC defects, which could be explained in the way that on one hand, Fe^2+^ ions are competing with PEC defects on releasing electrons to conduction, and Fe^2+^ ions hold the advantage, thus, much more electrons were captured by Q^3^ units resulting in the formation of PEC defects. On the other hand, more PEC defects were generated for the purpose of balancing the oxidized (Fe^2+^)^+^ HC defect. Therefore, the concentration levels of PEC defects increased, especially for the PO_2_-EC defect.

**Figure 9:**
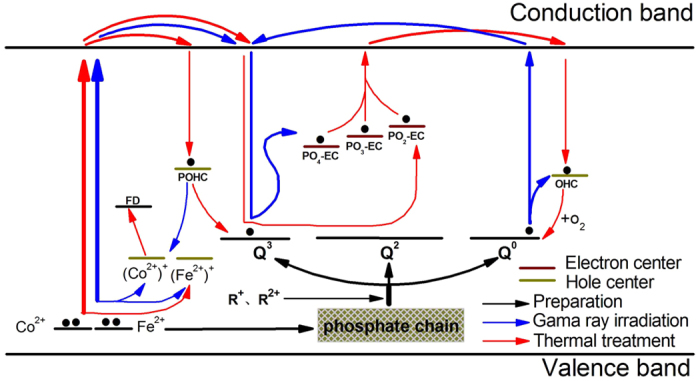
Schematic representation of the Gamma radiation and thermal treatment process in the fluoride containing phosphate based glasses (R^+^: alkali metal cations. R^2+^: alkaline earth metal cations).

To fully understand the generation of the induced defects in these investigated gamma irradiated phosphate based glasses and their healing behavior upon the heat treatment at ambient conditions, we propose a model to explain the results of radiation induced absorption and bleaching experiments as shown in Fig. 9. Firstly, several phosphate units, i.e. Q^0^, Q^2^ and Q^3^ are formed in the phosphate based glasses containing the alkali and alkaline earth metal cations (R^+^, R^2+^). And the reducing glass melting atmosphere causes the formation of OHC, PO_3_-EC, PO_4_-EC and PO_2_-EC defects during the glass preparation process. Besides, some intentionally doped iron and cobalt ions may enter the glass skeleton structure, as shown by the black arrow in Fig. 9. Under gamma radiation, the ground-state electrons of Co^2+^ and Fe^2+^ ions were photo-ionized to the conduction band, then (Co^2+^)^+^ and (Fe^2+^)^+^ HC defects form in the two kinds of doped glasses. Meanwhile, Q^0^ was also photo-ionized then OHC defect generated, which have contributed to the increased absorption bands as shown in Fig. 2. Parts of conduction band electrons were subsequently captured by Q^3^ and transformed into PO_3_-EC, PO_4_-EC and PO_2_-EC defects, as shown by the blue arrow in Fig. 9. In addition, extrinsic HC including (Co^2+^)^+^ and (Fe^2+^)^+^ defects may replace intrinsic POHC, which result in the decreased concentration of POHC defect. In the case of heat treatment at ambient atmosphere, some Fe^2+^ were oxidized to (Fe^2+^)^+^ and this contributed to the increased concentration of (Fe^2+^)^+^ defect. Meanwhile, thermal energy may induces the release of trapped electrons in PO_3_-EC, PO_4_-EC and PO_2_-EC defects through the conduction band, and then recombines with POHC defects and form Q^3^ units. The remainder electrons were captured by OHC defects which lie in oxygen rich atmosphere, leading to the formation of Q^0^ units, which result in the decrease of OHC defect concentration. Therefore, defects formation includes the healing of defects by recombination of HC with EC defects^19^. With regard to FD centers, on the one hand, gamma radiation may cause breaking of chemical bonds associated with F and lead to the increase of FD defects. On the other hand, a significant transformation of Co^2+^ and (Co^2+^)^+^ defects into FD center defects can be deduced from Fig. 8, and the effects become more apparent at higher thermal treatment temperatures.

We have quantitatively estimated the variation in the concentration of defect in fluoride-containing phosphate based glasses through the analysis of the peak-differentiation-imitating of the absorption spectra and corresponding peak intensity of the defect. Besides, we observe the ESR spectra to determine the presence of similar defect. Based on the above results and discussion, it is reasonable to be a possible new way that reducing atmosphere could reduce the POHC and OHC defects in the fluoride-containing phosphate based glasses during the preparation process. Besides, the post-heat treatment could also further eliminates the induced defects caused by irradiation of gamma ray, and promotes the transformation of extrinsic defects including Co^2+^ and (Co^2+^)^+^ defects into FD center defects, while other intrinsic defects including PO_3_-EC, PO_4_-EC defects are remaining stable.

We have studied the generation of the defects in the gamma ray irradiated fluoride containing phosphate based glasses and their defects healing behavior upon the heat thermal treatment at ambient conditions. Gamma radiation causes the increase of various defects in these glasses that contribute to the large absorption in UV region. The thermal bleaching experiment results indicate the heat treatment bleached gamma radiation induced POHC and OHC defects to some extent due to the release and capture the electrons in conduction band. In further, the heat treatment near the glass transition temperature will contribute to the defects healing behavior more effectively.

## Methods

### Glass preparation, gamma radiation and post-heat treatment

The undoped and doped (iron (0.58 wt%) and cobalt (0.05 wt%) ions single-doped) phosphate based glasses with a low content of fluoride were prepared from high purity raw materials (≥99.9%, Sigma Aldrich Inc.) with a weight composition (wt%) of Li_2_O (0.5–2), K_2_O (3–5), MgO (3–5), BaO (7–10), Al_2_O_3_ (8–11), P_2_O_5_ (59–64), YF_3_ (0–1) and LaF_3_ (0–2). The iraurite crucible containing the mixture and reducing agent was placed in an electric furnace under a reducing atmosphere with continuous flowing of 95% N_2_ + 5% H_2_. In contrast, the undoped glass with the same composition was intentionally made in an ambient air atmosphere in a silica crucible. The glass melts were refined and homogenized through a mechanical stirring process at 1200 ^o^C for 12 h and then cast into a copper mold preheated at 300 ^o^C. The molded samples were annealed at 400 ^o^C (near the glass transition temperature) through a precision annealing process with a cooling rate of −1 ^o^C/h from 400 ^o^C to 300 ^o^C, followed by −3 ^o^C/h from 300 ^o^C to 150 ^o^C. After annealing, all the glass samples were cut and polished using zirconia (ZrO_2_) micro-particles. In order to study the evolutionary mechanism of defects in these phosphate based glasses, all of the glass samples (with a thickness of 10 mm and 1.5 mm, respectively) were exposed to gamma radiation using a ^60^Co source at 5.2, 7.8 and 10.4 rad (Si)/s, respectively, to accumulate absorbed doses of 10, 15 and 20 krad (Si) (The SI unit of an absorbed dose is 1 Gray = 100 rad.). The samples with thickness of 10 mm were firstly irradiated for a general comparison of their transmittance decline in the UV-VIS range due to defects induced absorption. Further experiments were carried out on the samples with a thickness of 1.5 mm was to get better information about formation and bleaching of these defects having absorption in the near UV range. The irradiated glass samples with a thickness of 1.5 mm were heat treated at 90 ^o^C, 190 ^o^C, 290 ^o^C and 390 ^o^C at ambient conditions, respectively, to investigate the healing behavior of the radiation induced defects upon heat treatment temperatures.

### Characterization methods

The transmission spectra were recorded with a UV-VIS-NIR spectrophotometer (Shimadzu UV-3101) in the range of 200–700 nm. The separation of the absorption spectra was carried out using Gaussian peak fitting method^1^,^6^, and all the fitted absorption-peak represents the parameters Adj R-Square ≥0.9998 and reduced Chi-Sqr ≤ 0.0011. EPR measurements of the glass samples before and after gamma radiation were conducted at 100 K using a Bruker EMX spectrometer, operated at an X-band frequency of 9.677 GHz with field modulation 100 kHz. The microwave power used was 20 mW. Raman spectra were collected with a Jobin-Yvonne LabRam microscope with a 514 nm laser excitation in the range of 100–1500 cm^−1^.

## Additional Information

**How to cite this article**: Wang, P. F. *et al.* Evolutionary mechanism of the defects in the fluoride-containing phosphate based glasses induced by gamma radiation. *Sci. Rep.* **6**, 18926; doi: 10.1038/srep18926 (2016).

## Figures and Tables

**Table 1:**
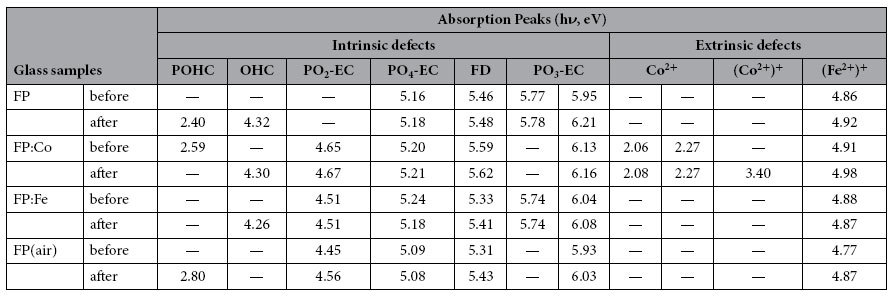
Absorption peaks identified from the Gaussian peak fitting of the absorption spectra of FP, FP: Co, FP: Fe and FP (air) before and after gamma radiation.
